# Immobilization of Magnetic Nanoparticles onto Amine-Modified Nano-Silica Gel for Copper Ions Remediation

**DOI:** 10.3390/ma9060460

**Published:** 2016-06-09

**Authors:** Marwa Elkady, Hassan Shokry Hassan, Aly Hashim

**Affiliations:** 1 Fabrication Technology Department, Advanced Technology and New Materials Researches Institute, City of Scientific Researches and Technological Applications, New Borg El-Arab City, Alexandria 21934, Egypt; lionth@hotmail.com; 2 Chemical and Petrochemical Engineering Department, Egypt-Japan University of Science and Technology, New Borg El-Arab City, Alexandria 21934, Egypt; 3 Electronic Materials Researches Department, Advanced Technology and New Materials Researches Institute, City of Scientific Researches and Technological Applications, New Borg El-Arab City, Alexandria 21934, Egypt

**Keywords:** nano-magnetic silica gel hybrid, amine-functionalized silica gel, copper remediation, equilibrium and kinetics modelling

## Abstract

A novel nano-hybrid was synthesized through immobilization of amine-functionalized silica gel nanoparticles with nanomagnetite via a co-precipitation technique. The parameters, such as reagent concentrations, reaction temperature and time, were optimized to accomplish the nano-silica gel chelating matrix. The most proper amine-modified silica gel nanoparticles were immobilized with magnetic nanoparticles. The synthesized magnetic amine nano-silica gel (MANSG) was established and characterized using X-ray diffraction (XRD), transmission electron microscopy (TEM), scanning electron microscopy (SEM), Fourier transform infrared (FTIR), thermal gravimetric analysis (TGA), differential scanning calorimetry (DSC) and vibrating sample magnetometry (VSM). The feasibility of MANSG for copper ions’ remediation from wastewater was examined. MANSG achieves a 98% copper decontamination from polluted water within 90 min. Equilibrium sorption of copper ions onto MANSG nanoparticles obeyed the Langmuir equation compared to the Freundlich, Temkin, Elovich and Dubinin-Radushkevich (D-R) equilibrium isotherm models. The pseudo-second-order rate kinetics is appropriate to describe the copper sorption process onto the fabricated MANSG.

## 1. Introduction

Water pollution by trace heavy metals is well known as a serious environmental and public problem. Where the increasing in industrial activities has caused many water bodies to receive loads of heavy metals that exceed the maximum permissible limit for wastewater, discharge is designed to protect the environment, humans and animals. These metals, in addition to their high toxicity, do not have any tendency to be degraded or destroyed. Accordingly, in order to provide long-term high quality water or to enable water recycling, there has been research into alternative remediation processes involving filtration, chemical precipitation, solvent extraction, electrolysis, ion exchange, electrochemical deposition and membrane process. However, these methods are either inefficient or expensive, especially when the concentration of the heavy metal ion is low [1].

Solid phase extraction (SPE) has commonly been used as a technique for pre-concentration/separation of various inorganic and organic species. It is used to enhance the selectivity and sensitivity of the method, as it allows for discriminatory binding of the analyte to a solid support, where it will be accumulated and subsequently eluted with a small volume of solvent. This technique has the advantages of a higher enrichment factor, the absence of emulsion, safety with respect to hazardous samples, minimal costs due to low consumption of reagent, being environmentally friendly, flexibility and easier incorporation into automated analytical techniques [2,3]. For this purpose, new sorbent materials, such as polymeric resins, activated carbon, naphthalene and silica gel, have been developed for more effective extraction. However, some of these sorbents, especially the organic ones, such as polymeric resin, suffer from a number of drawbacks, such as slow kinetics, irreversible adsorption of organics, swelling, sensitivity towards many chemical environments and loss of mechanical stability in modular operation.

These problems lead to developing inorganic sorbent nanomaterials as alternatives for polymeric resin; where most of these nano-inorganic materials are distinguished by their good selectivity, no swelling, rapid sorption of metal ions and good mechanical stability [4]. The development of novel and cost-effective nanomaterials for environmental remediation, pollution detection and other applications has attracted considerable attention. Recent advances suggest that many of the issues involving water quality could be resolved or greatly ameliorated using nanoparticles, nanofiltration or other products resulting from the development of nanotechnology.

One of the potential and inexpensive inorganic sorbent nanomaterials that could be used in water treatment is nano-silica gel. In addition to its high mechanical, thermal and chemical resistance, it also is distinguished by its local availability and has many microsized pores on the surface to induce and adsorb various molecules into the pores [5,6]. These characteristic features provide silica gel materials the possibility of their surface modification or to be coated with an impregnation medium or reagent. Subsequently, they can be chemically modified with different functional groups either with inorganic or organic functionalities to improve their removal performance. These modified materials have been commonly used in various areas, most notably in the separation and preconcentration of trace metal ions from aqueous systems. With respect to the immobilization (modification or fictionalization), reactions on silica gel materials are relatively simple and show fast kinetics in metal ions’ uptake [4,7]. Therefore, silica gel will be functionalized with an amine group as an effective functional group to chelate copper ions from polluted waste water. The factors that affect the silica gel functionalization process will be optimized to attain silica gel with a high affinity for copper ion sorption. The most proper functionalized silica gel that poses the highest copper sorption affinity will be immobilized with nano-magnetite to fabricate a novel nano-magnetic silica gel hybrid material. This magnetic nano-hybrid is characterized by its unique and useful magnetic property that facilitates its separation from the treatment media using an external magnetic field. Moreover, the copper sorption process using the fabricated magnetic amine silica gel nano-material (MANSG) will be monitoring as a function of the processing parameters, such as copper concentration, dosage of adsorbent material, pH of copper solution and temperature. Finally, different theoretical models will be proposed for describing the equilibrium and kinetic copper sorption process onto the MANSG.

## 2. Materials and Methods

### 2.1. Materials

A stock solution of copper of 5000 mg/L was prepared by dissolving copper(II) chloride 2-hydrate (170.48 g/mol, Riedel, Germany) in distilled water. Different concentrations ranging between 10 and 5000 mg/L were prepared from the copper stock solution. Before mixing the adsorbent material with the copper-polluted waste water, the pH of each solution was adjusted to the required value with dilute solutions from 0.1 N HCl and 0.1 N NaOH. All of the other chemicals utilized for the non-magnetic amine-functionalized silica gel synthesis process were of analytical reagent grade.

### 2.2. Methods

#### 2.2.1. Preparation of Amine-Functionalized Nano-Silica Gel

Firstly, the raw silica gel of a micro-scale diameter (20 µm) was activated through mixing 10 g of silica gel (GF254; Fluka, Switzerland) with 50 mL hydrochloric acid (4 M) (37%; 36.46 g/mol, Riedel, Germany) under stirring for 4 h at a 70 °C refluxing temperature. The produced activated silica cake was washed several times with distilled water then filtered under vacuum suction. The produced white precipitate was dried at 150 °C for 5 h. 

In order to chemically modify and functionalize the previously-activated silica gel, it was co-condensated with 3-amino propyl triethoxy silane to be functionalized with the amine group. One gram from the dried powdered material was mixed with various volumes of toluene solution (0.5, 1, 2, 4 and 6 mL/g) and a specific volume of 3-amino propyl triethoxy silane (0.05, 0.1, 0.25, 0.4 and 0.6 mL/g) under a refluxing system in the presence of argon. The refluxed reaction mixture stands under different temperatures (40, 50, 60, 70 and 80 °C) for different reaction time intervals (2, 4, 6, 12, 19 and 22 h). The modified silica gel produced was filtered under vacuum filtration and washed with toluene and acetone, then dried in a vacuum oven for 3 h at 70 °C. The copper sorption efficiency of all synthesized samples at the various studied preparation conditions was screened to attain the most efficient amine-functionalized sample that records the maximum copper sorption capacity. 

#### 2.2.2. Preparation of Magnetic Amine-Functionalized Nano-Silica Gel

**Figure 1:**
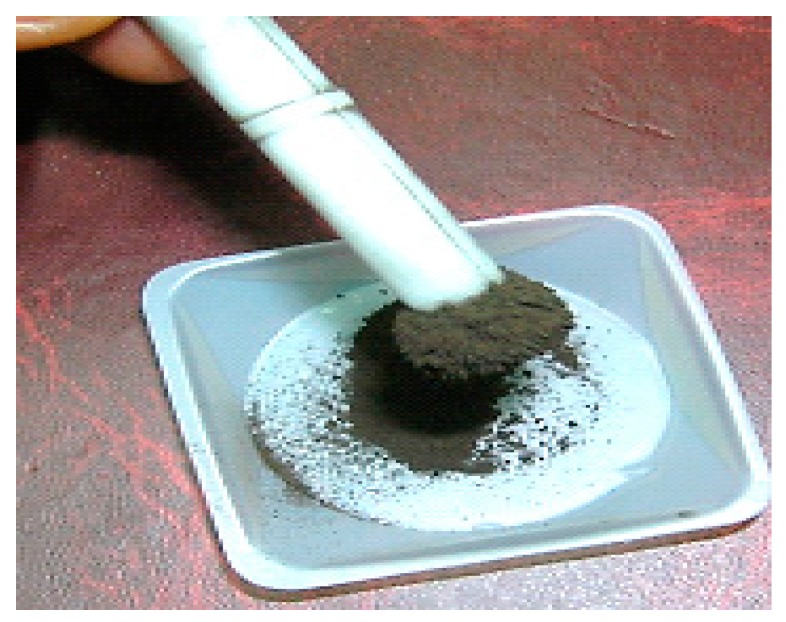
Magnetic performance of the fabricated magnetic amine-functionalized silica gel nano-hybrid material (MANSG).

The most proper amine-functionalized silica gel that recorded the highest copper sorption capacity was immobilized with nano-magnetite using the co-precipitation technique to attain a magnetic nano-hybrid. Three grams of dry amine-functionalized silica gel were suspended in 400 mL of ferric and ferrous salt solution. This solution was composed of 15 mmol ferric chloride and 10 mmol ferrous sulfate. The previous suspension was heated under a constant mixing rate (400 rpm) using a magnetic stirring hot plate at 60 °C in the presence of N_2_. Then, 3 mol/L sodium hydroxide solution was added dropwise to the previous heated suspension under continuous heating and stirring in the presence of a N_2_ atmosphere until the complete addition of the sodium hydroxide solution. A black precipitate appeared during the NaOH addition, converting the white suspension (of the chemically-modified silica gel) into the black precipitate of the magnetic silica gel hybrid. This black precipitate was filtered and dried overnight under vacuum drying at 60 °C. After complete dryness of the black precipitate, its magnetic performance was tested using a magnetic bar; the magnetization of the prepared magnetic composite is evident from Figure 1, where the black powder material has a high attraction affinity toward the magnetic field.

#### 2.2.3. Characterization of the Magnetic Amine-Functionalized Nano-Silica Gel

Different characterization techniques have been used to identify the fabricated MANSG’s properties. X-ray powder diffraction (XRD) was carried out using an X-ray diffractometer (Schimadzu-7000, Kyoto, Japan) with a CuKα radiation beam (λ = 0.154060 nm). The fine black powdered sample was packed into a flat aluminum sample holder. Data were collected between 10° and 80° in 2θ. In order to assign the characteristic functional groups present in the fabricated MANSG hybrid material, the dried black powder was analyzed as KBr discs using Fourier transform infrared (FTIR) (Shimadzu FTIR-8400 S, Kyoto, Japan) in the frequency range 600–4000 cm^−1^ at room temperature. The morphological features of the adsorbent material were determined using a transmission electron microscope (JEOL JEM 1230, Tokyo, Japan). The carbon-coated copper grid was immersed into the ultrasonic homogenized fully-dispersed suspension composed of MANSG nano-powder and ethanol. Then, the coated grid was dried at room temperature to be examined under an acceleration voltage of 100 kV. The thermal stability of the magnetic amine-functionalized nano-silica gel was evaluated by a thermogravimetric analyzer (Shimadzu TGA-50, Kyoto, Japan) and a differential scanning colorimeter (Shimadzu DSC-60, Kyoto, Japan). The measurements were carried out with a heating rate of 20 °C/min under N_2_ flow to avoid thermal oxidation of the powder samples, starting from the ambient condition up to 800 °C. The magnetization properties of the fabricated MANSG hybrid were determined at room temperature up to a maximum magnetic field of 900 Tesla using a vibrating sample magnetometer (VSM, Dexing, Model: 250, Lake Zurich, IL, USA). 

#### 2.2.4. Adsorption Technique for Copper Remediation from Polluted Water Using the MANSG Nano-Hybrid

The batch experiments were carried out using a digital heating controlled magnetic stirrer (J.P. Selecta, Barcelona, Spain). The copper sorption process was evaluated as a function of the variation in the processing parameters of the nano-magnetite immobilized amine-modified silica gel (MANSG). A known volume (25 mL) from metal solutions with varying initial concentrations (10–5000 ppm) was taken in a 100-mL conical glass stopper. This solution was shaken with variant doses from the adsorbent material (5–60 g/L) at different contact time periods (5–240 min) at various agitation speeds (0–400 rpm) under different solution temperatures (25–85 °C) and different solution pH (2–12). The residual copper ion concentration in the solution after the treatment process was analyzed using an inductive coupled plasma mass spectrophotometer (ICP-AES, Santa Clara, CA, USA). All of the experiments were carried out in duplicate, and mean values are presented. The copper ion measurements on the ICP equipment were repeated three times to obtain an accurate copper concentration. The percentage error in the measurement of copper ion concentration is ±0.1. The mean copper concentration values were obtained and used for the calculation of the percentage of ion removal by the MANSG from the following Equation (1):

(1) $$\%R=(\frac{C_{O}-C}{C_{O}})100$$

where *R* is the ion removal, *C_o_* is the initial concentration of the metal ions in solution and *C* is the final metal ion concentration in aqueous solution after solid phase separation. 

In order to elucidate the uptake capacity of the metal ion, the up take amounts per gram of MANSG were evaluated from the change in solution concentration using Equation (2):

(2) $$Q(\text{mg/g})=\frac{V({\text{C}}_{O}-\text{C})}{M}$$

where *Q* is the uptake capacity (mg/g), V is the volume of the solution (mL) and M is the mass of the solid material (g).

#### 2.2.5. Desorption of Copper Ions from the Contaminated MANSG Nano-Hybrid after the Treatment Process

In order to regenerate MANSG that was contaminated with the adsorbed copper ions after the treatment process, copper ions’ desorption was conducted by batch experiments. A 1-L solution containing 1000 ppm of copper ions was treated with 20 g/L of MANSG under a 200-rpm mixing speed for 90 min. After finishing the treatment period, the copper-contaminated MANSG material was separated from the treatment media by a magnetic field (magnetic bar), and the residual filtrate was analyzed for copper ions. Copper containing the MANSG adsorbent material was transferred to another beaker and stirred for 90 min at 200 rpm with 1 L of 0.02 N EDTA to determine desorbed copper ions from MANSG. Liquid samples were withdrawn at predetermined time intervals and analyzed for stripped copper ion concentration.

## 3. Results and Discussion

### 3.1. Preparation of the Amine-Functionalized Nano-Silica Gel Silica Gel Activation Process

**Scheme 1:**
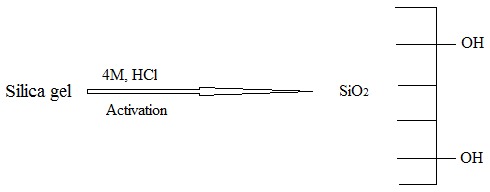
Chemical activation of silica gel.

The silica gel activation process represented a preliminary step in silica gel functionalization. Silica gel was immersed in hydrochloric acid solution to remove the trace impurities associated with powdered material, then dried to evaporate the water molecules that bonded with the silanol group (Si-OH) inside the silica gel matrix through hydrogen bonding. Accordingly, these free silanol groups inside the silica gel matrix give the opportunity for the silica matrix to form a covalent bond with new functional groups from the reaction media. Moreover, this loss of water molecules from silica gel improves its surface area and its pores, which enhances the physical adsorption, chemical adsorption and capillary condensation processes, becoming more efficient and effective, as illustrated Scheme 1; on the other hand, this drying process is important because much water is left in the mesopores, which may cause an oligomerization of the silanes and pore blocking [8,9].

### 3.2. Optimization of the Chemical Modification Process of the Activated Silica Gel

**Scheme 2:**
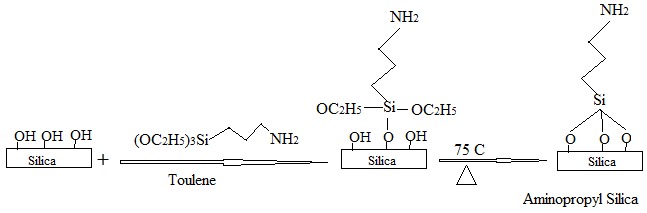
Chemical functionalization of activated silica gel with silanol groups.

Activated silica gel was modified chemically using 3-amino propyl triethoxy silane (APTS). During the modification, silane coupling agents that attain the amine functional groups are substituted by silanol groups present on the silica surface according to the mechanism of electrophilic proton substitution, as illustrated in Scheme 2. In this mechanism, the contribution of the centers of this surface has been taken into account [10].

Different factors will influence the chemical substitution process of the activated silica gel, such as the reaction time, the reactant concentration and the reaction temperature. These factors will be optimized to functionalize the activated silica gel with the amine functional group.

**Figure 2:**
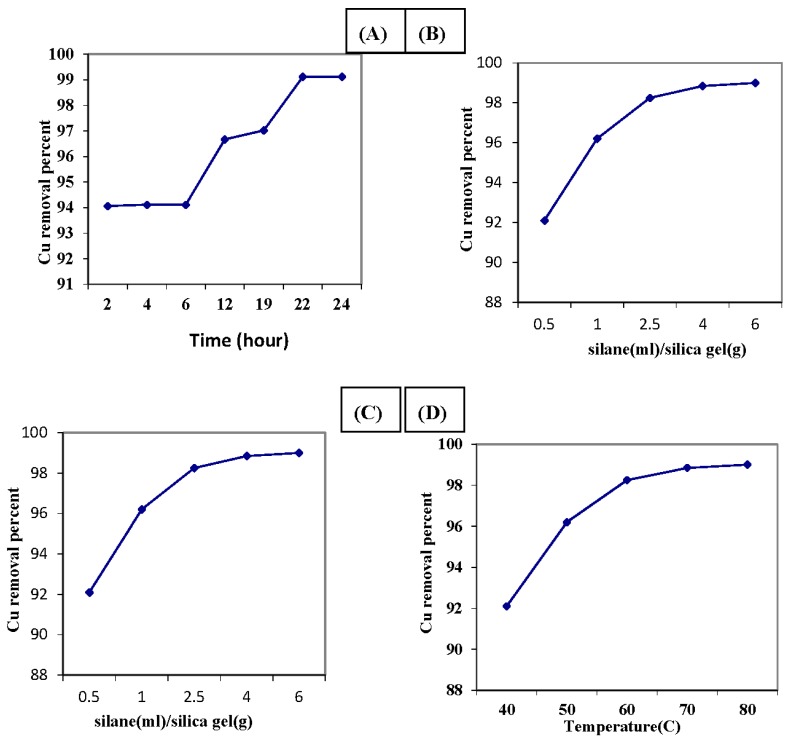
Effect of chemical modification parameters on amine-functionalized silica gel copper sorption capacity. (**A**) Effect of reaction time; (**B**) effect of the variation of the solvent to silica gel ratio; (**C**) effect of the variation of the propyl triethoxy silane (APTS) to silica gel ratio; (**D**) effect of the reaction temperature. Solution concentration of copper ion = 100 ppm; amount of ANSG = 0.2 g; agitation time = 90 min; agitation speed = 200 rpm; solution volume = 25 mL; pH = 7.

Firstly, the effect of reaction time on the sorption efficiency of the produced ANSG was monitored to determine the copper removal capacity of the modified silica gel produced at each studied reaction time interval (Figure 2A). It was elucidated by this figure that as the reaction period increased, the copper removal capacity of the produced material was incremented. This may be related to the short time reaction intervals leading to an incomplete chemical modification process for the activated silica gel. Consequently, it was suggested that the optimum reaction time for the chemical medication of silica gel is 22 h. This reaction time interval is compatible with many researchers’ results [11], where they stated that 24 h is a sufficient period for completing the chemical modification process of silica gel [9].

The solvent has a critical role in the reaction rate because solvents may or may not surround a nucleophile, thus hindering or not hindering its approach to the carbon atom [12]. Therefore, the ratio of toluene solvent to silica gel will be firstly optimized to adjust the reactant ratio of the reaction mixture to prepare the most efficient sorbent material for copper sorption at the predetermined 22-h reaction time. The effect of the changing toluene to silica gel concentration from 5–60 mL/g at a fixed 3-amino propyl triethoxy silane (APTS) concentration was monitored for the copper sorption affinity of the produced ANSG. Figure 2B demonstrates that the increment in the toluene concentration improves the copper removal affinity of the produced ANSG. This may return to the competition between both the solvent and organic groups for the reaction with the surface silanol groups present in the pores by means of an interaction with APTS generated through hydrogen bonds during the functionalization process. As the solvent concentration increased, a preference of hydrogen bonding occurs between the central region of APTS and the silanol groups of silica gel, rather than the weak van der Waals forces on the periphery. As such, the molecules of the APTS are more thermodynamically stabilized by hydrogen bonds than by the van der Waals interactions within the toluene solvent [13]. Finally, it can be concluded that the optimum toluene to silica gel ratio was recorded to be 20 mL/g.

Based on this optimum toluene to silica gel ratio, the most proper amount of APTS-functionalized agent utilized for grafting the activated silica gel to produce a highly efficient copper sorption adsorbent material will be determined. The studied reaction ratio of APTS to silica gel in the reaction mixture was varied from 0.5–6 mL/g using 20 mL/g toluene solvent. The influence of APTS concentration on the copper sorption performance of the produced sorbent materials was illustrated in Figure 2C. It was evident from this figure that the increment in the amount of APTS in the reaction mixture was associated with improvement in the copper removal capacity of the produced modified silica gel. This observation may return to incrementing in the degree of silica gel functionalized with amine groups as the amount of APTS coupling agent increased [13]. That is, it improves the adsorption capacity of modified silica gel for copper removal. Accordingly, the optimum APTS to silica gel ratio was determined to be equal to 2.5 mL/g.

The effect of the reaction temperature on the silica gel amine grafting process was elucidated using all of the predetermined optimum silica gel modification process conditions. The copper removal capacity of the produced modified silica gel at different reaction temperatures was investigated at Figure 2D. It is clear that the reaction temperature has a positive effect on the copper sorption affinity for the produced modified silica gel. This may be in regards to the fact that the increase in the reaction temperature favors the diffusion rate of reactants. That is, it improves the reaction rate and subsequently increases the degree of silica gel grafting by amine groups, which enhance the adsorption behavior of the modified silica gel to increase its affinity for copper removal. Accordingly, the optimum reaction temperature was 70 °C for the chemical modification process, where it produces adsorbent material with a 98% copper removal affinity.

According to the optimized preparation conditions, the most proper amine-functionalized silica gel (ANSG) that attains the optimum copper decontamination of 98% was synthesized through the co-condensation of 1 g silica gel with 20 mL toluene and 2.5 mL silane for 22 h at 70 °C. This functionalized silica gel was immobilized with nano-magnetite to attain nano-magnetic amine-functionalized silica gel using a co-precipitation technique. 

### 3.3. Characterization of the Magnetic Amine-Functionalized Nano-Silica Gel

The properties of MANSG were mostly compared to its parent amine-functionalized silica gel (ANSG) before immobilization to record the main difference between the two matrices.

**Figure 3:**
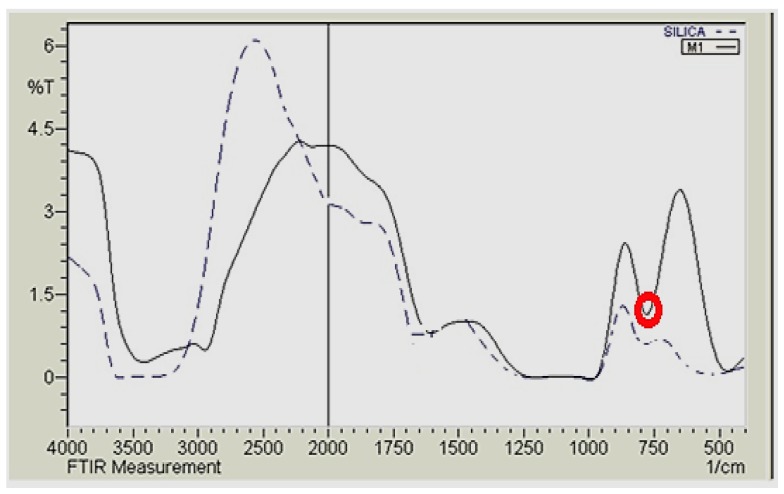
FTIR for amine-functionalized (dashed line) and magnetic immobilized functionalized (straight line) silica gel.

The FTIR spectra of both amine-functionalized silica gel and its magnetic immobilized matrices are investigated in Figure 3 to determine their main functional groups. Comparing the two spectrums, they show relatively similar spectra and reveal the presence of an amine-functionalized band (NH_2_) at around 1650 cm^−1^. Additionally, both matrices demonstrated the stretching band at 3200 cm^−1^ for functionalized silica gel that shifted to 2943.17 cm^−1^ for the magnetic silica gel, which was assigned to a C-H weak band of the carbon chain of the pendant group attached to the inorganic silica matrix [14]. The shoulder band at 960 cm^−1^ present in the FTIR spectra of these matrices is associated to SiOH vibrations and composed of Si-O-H stretching. A new observed band was recorded in the magnetic immobilized silica gel spectrum around 750 cm^−1^, which is responsible for the vibration of the M-O-M of the magnetite particle immobilized onto the silica gel matrix [15]. These FTIR results confirm the amine functionalization of silica gel, as well as the immobilization of magnetite onto the functionalized silica gel.

**Figure 4:**
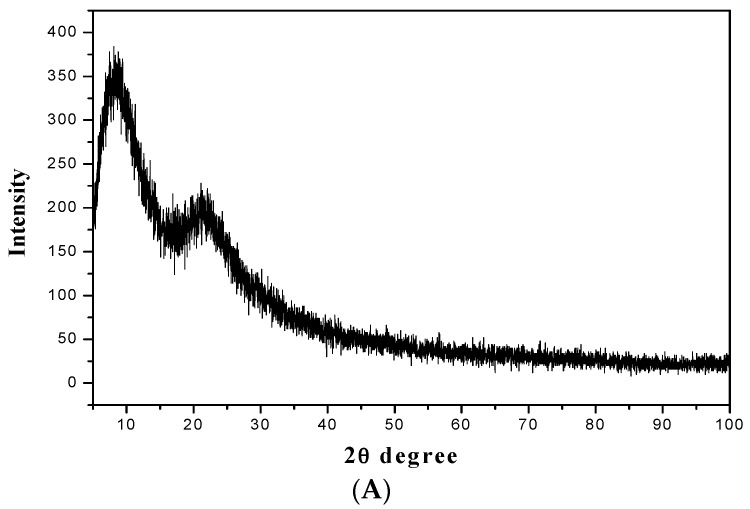

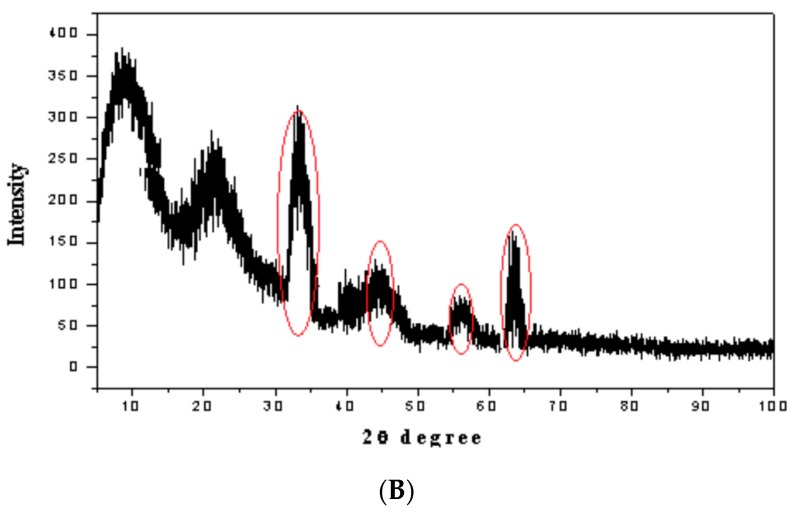
XRD spectrums of (**A**) amine-functionalized silica gel (ANSG) and (**B**) magnetic amine-functionalized silica gel (MANSG).

Figure 4 exemplifies the XRD patterns of the mesoporous materials of the amine-functionalized silica gel (ANSG) and its magnetic matrices (MANSG). The two patterns appear to consist of a broad peak in a low 2θ angle range, which suggests the presence of ordering mesoporous structure materials. Meanwhile, the ANSG pattern (Figure 4A) displayed a well-resolved pattern at low 2θ values with a sharp diffraction peak at 8.2° and a less intensive peak at the angle of 23° associated with (370), (222) reflections, respectively. The absence of peaks at a higher angle confirms that the silica layer has an amorphous structure [14]. On the other hand, noticeable characteristic peaks were observed at high 2θ values for the XRD pattern (Figure 4A) of magnetic-functionalized silica gel (ANSG) that explored the crystallinity of this matrix compared to the functionalized silica gel. Figure 4B shows the sum of reflection intensities (red highlighted) at 2θ of 35.5°, 43°, 57° and 63°, which correspond to the (311), (400), (511) and (440) planes of the cubic crystal of iron oxide Fe_3_O_4_, which confirmed by standard data for magnetite (Ref: 04007-1060) [16]. These peaks confirm the presence of crystalline magnetite nanoparticles. However, the intensity of the immobilized magnetite characteristic peaks is not high enough compared to the pure magnetite reference (Ref: 04-007-1060). This may be in regards to the incorporation of some of magnetite nanoparticles within the pores of the silica gel mesoporous structure [16].

**Figure 5:**
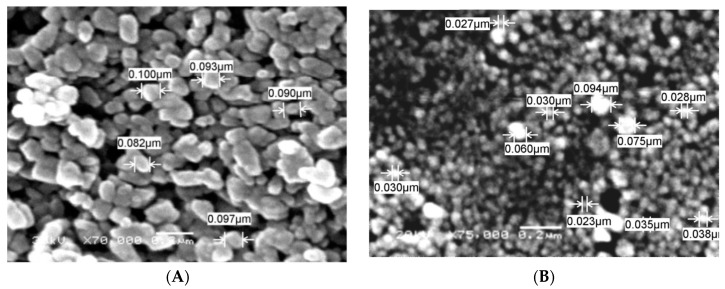
SEM morphological structure of (**A**) amine-functionalized silica gel (ANSG) and (**B**) magnetic amine-functionalized silica gel (MANSG).

**Figure 6:**
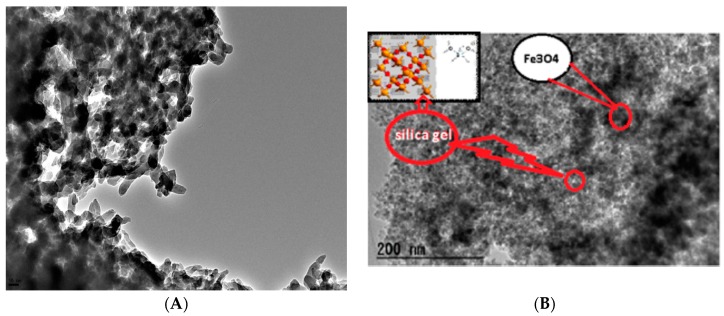
TEM imaging of (**A**) amine-functionalized silica gel (ANSG) and (**B**) magnetic amine-functionalized silica gel (MANSG).

Figure 5 represents the SEM micrographs of the amine-functionalized silica gel (Figure 5A) and its magnetic immobilized hybrid (Figure 5B). It was indicated that the particle morphology of the two matrices has spherical shapes with 100-nm and 50-nm average diameters for ANSG and MANSG, respectively. However, the raw silica gel before the chemical modification process has an average diameter equal to 20 µm (as indicated from its suppliers). Accordingly, these results designated that both the chemical functionalization and magnetite immobilization processes reduce the silica gel particle diameter from the micro-scale into the nano-scale. This may be owed to the severe chemical conditions of these processes that deform the silica gel bulk particles into nanospherical shapes as the action of chemical treatment processes under high temperature [17]. Moreover, the magnetic immobilized silica gel hybrid was subjected to further chemical treatment conditions of the magnetite immobilization process after the chemical modification process of silica gel functionalization that may explain the reduction of the nanosize of the magnetic matrices and its uniform shape compared to the amine-functionalized one. Moreover, the immobilization of magnetite nanoparticles onto the silica gel matrix is confirmed in Figure 6B. It was indicated from this figure that there are black spots of magnetic nanoparticles (highlighted in red) present over the silica gel matrix. This figure gives predictions that the mechanism of magnetite immobilization onto the silica gel matrix is based on the uniform dispersion of small nanoparticles of magnetite onto the porous structure of the functionalized silica gel matrix. This suggested mechanism is in accordance with other mechanisms described for the immobilization of magnetite onto the zeolite matrix to fabricate the magnetite zeolite nanocomposite [18]. This result confirms the formation of the magnetic amine-functionalized silica gel hybrid.

The particle size reduction and the regularity of the magnetic amine-functionalized silica gel are confirmed in Figure 6; where TEM imaging showed the reduction in the particles of the magnetic silica gel hybrid and its homogeneity in Figure 6B compared to the amine-functionalized one in Figure 6A. Moreover, the immobilization of magnetite nanoparticles onto the silica gel matrix is confirmed in Figure 6B, where the black spots of the magnetic nanoparticles (highlighted in red) were present over the silica gel matrix, confirming the formation of the magnetic amine-functionalized silica gel hybrid. Finally, the conversion of bulk silica gel to the nanosilica gel gives the prediction that the amine-functionalized and magnetic immobilized silica gel materials present the potential to be employed as adsorbents for metal ion uptake. Moreover, the coupling agents of amine group adsorbed on the surface of silica gel induce hydrophobicity at the material surface. Therefore, the silica gel matrices’ surface assumes an organophilic character that enhances their performance as adsorbent materials [10].

**Figure 7:**
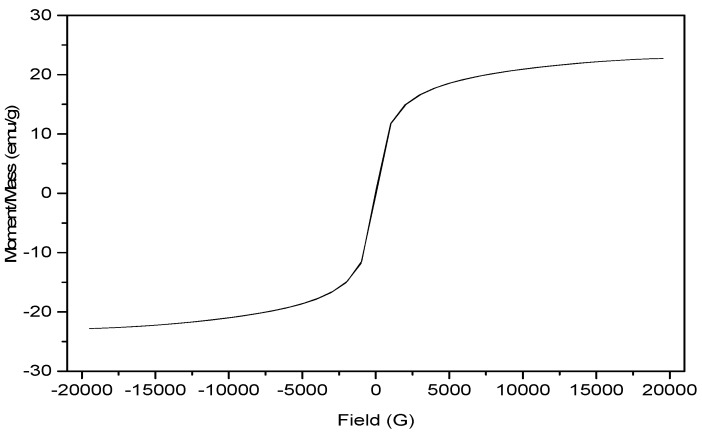
Isothermal M-H hysteresis curve of the magnetic amine-functionalized silica gel (MANSG).

In order to investigate the magnetic properties of the fabricated magnetic amine-functionalized silica gel hybrid (MANSG), the magnetization curve of the material at room temperature was elucidated at Figure 7. It was indicated that the magnetization hysteresis loop appears with an S-like shape; thus it does not display magnetic remanence. Accordingly, the magnetic silica gel was considered to be a super-paramagnetic matrix. It is observed from Figure 7 that the magnetization is completely saturated at a value of 22.775 emu/g for the MANSG matrix. The comparatively low value of the saturation magnetization (MS) may be in regards to the good dispersion of the magnetite nanoparticles in the silica gel matrices, which lead to the easiest formation of the magnetic domain [18]. It can be readily observed that the smaller particle sizes exhibit a smaller value of Ms, as expected, due to the surface disorder and modified cationic distribution [19]. 

**Figure 8:**
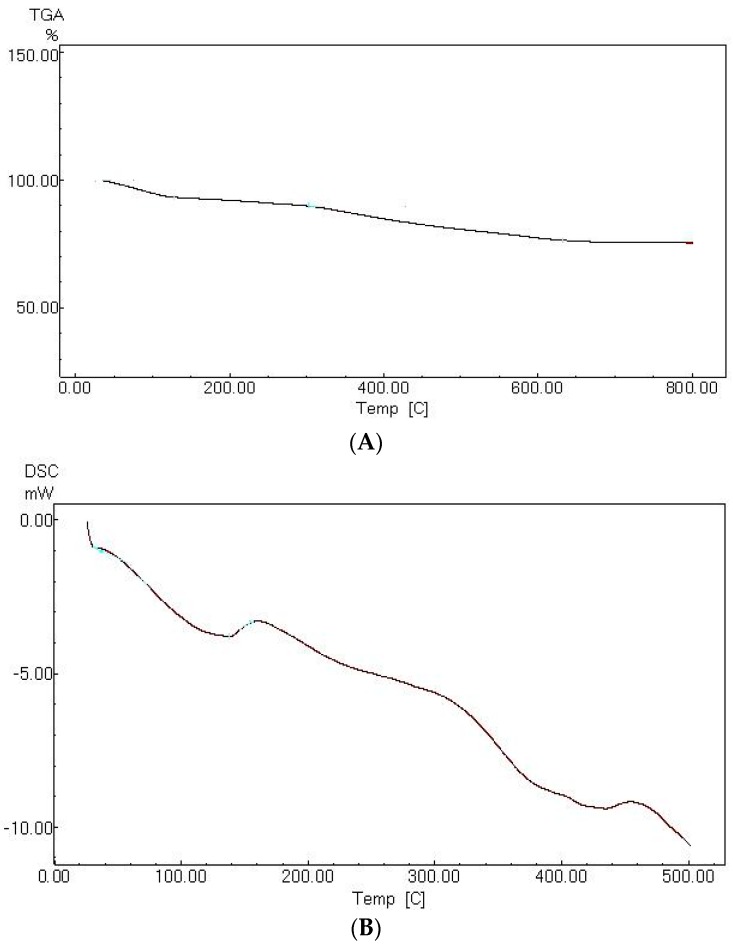
Thermal profile of magnetic amine-functionalized silica gel (MANSG): (**A**) TGA; (**B**) DSC.

The thermal profile of the fabricated MANSG hybrid is investigated in Figure 8. The resulting TGA material profile (Figure 8A) showed two main degradation steps at mid-points of 26–128 °C and 302–633 °C. The first gradual loss step assigned with a low percent of material weight loss equivalent to 7% corresponds to the loss of humidity and water contamination in the magnetic amine-functionalized silica gel matrix. The second degradation step represents the de-hydroxylation of the organic amine group, which was grafted onto the silica surface [20]. Generally, the total weight losses with respect to the surface water and amine group degradation of the magnetic hybrid matrix are less than 15%. These results indicate the high thermal stability of the fabricated MANSG hybrid material up to 800 °C.

The DSC pattern of the MANSG hybrid material (Figure 8B) verifies the previously discussed TGA results, where it shows two main endothermic peaks. The first broad endothermic peak represented around 100 °C was attributed to the heat losses due to the gradual loss of external water molecules from the material. This is followed by a narrow shoulder, which terminates in another broad peak at a temperature of around 300 °C, which may be ascribed to the combustion of the amine group grafted onto the silica gel surface [20].

### 3.4. Copper Sorption Profile of the Magnetic Amine-Functionalized Nano-Silica Gel

The copper sorption properties of the optimized amine-functionalized silica gel after nano-magnetite immobilization (MANSG) were investigated to explore the diversity of its behavior toward the copper ions.

**Figure 9:**
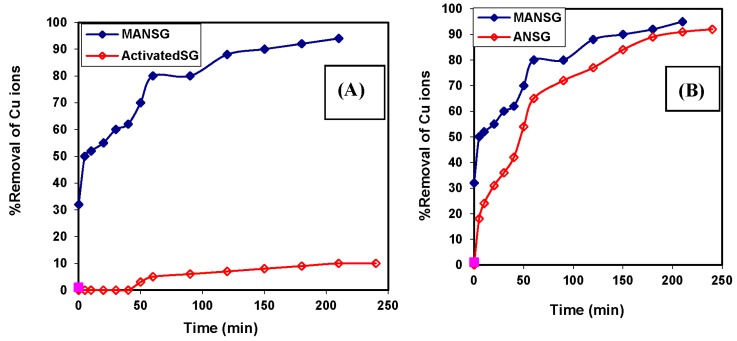
Comparable investigation of the influence of contact time on copper sorption process: (**A**) for both MANSG and its parent activated silica gel (Activated SG); (**B**) for both MANSG and its parent amine-functionalized nano-silica gel (ANSG). Solution concentration of ion = 1000 ppm; amount of adsorbent = 0.25 g; agitation speed = 200 rpm; solution volume = 25 mL; pH = 7.

Firstly, the copper sorption profiles of both magnetic amine-functionalized nano-silica gel hybrid (MANSG) and its parent activated silica gel before amine functionalization were compared to investigate the effect of amine group functionalization on the copper sorption process. On the other hand, a comparable investigation between the copper sorption behavior of both the magnetic hybrid (MANSG) and amine-functionalized silica gel before magnetite immobilization (ANSG) showed the effect of the magnetite immobilization process. Figure 9A implies that the copper decontamination of activated silica gel improved from 10%–98% for the fabricated MANSG. This confirms that the amine functional group has a great influence on the copper removal process of the magnetic silica gel hybrid. Figure 9B evidences that the magnetic hybrid (MANSG) attains 80% copper removal compared to 72% copper removal using ANSG. Accordingly, the presence of magnetite in the amine-functionalized silica gel slightly increases the capacity of the hybrid for the copper sorption process. Finally, it was indicated from the comparable investigation (Figure 9) that both MANSG and ANSG have a high copper sorption efficiency and compared to their parent material of activated silica gel. Furthermore, the sorption process onto these materials occurred rapidly and reached equilibrium after 90 min compared to the activated silica gel. This is likely due to the higher accessibility of copper analyte through the amine group present in both the amine-functionalized silica gel and its magnetic hybrid matrices [21]. The copper decontamination process onto both MANSG and ANSG showed a fast rate of sorption during the first hour of the sorbate-sorbent contact, and the rate of copper percent removal became almost insignificant due to the quick exhaustion of the sorption active sites. Moreover, the rate of the percent of copper ion removal was higher in the beginning due to the larger surface area of the adsorbent being available for the sorption of the ions [22].

**Figure 10:**
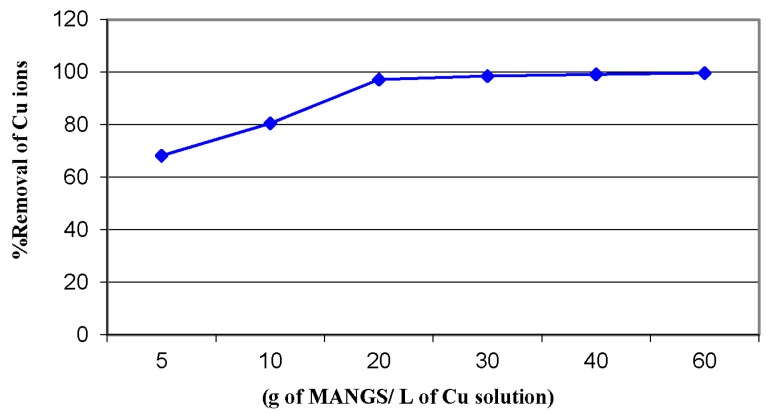
Effect of the MANSG dose on the copper sorption process (solution concentration of ion = 1000 ppm; time = 90 min; agitation speed = 200 rpm; solution volume = 25 mL; pH = 7).

The effect of adsorbent dosage on the adsorption process was examined at the equilibrium time. Figure 10 indicates that the percentage of copper ion removal increased from 62%–99.9% as the MANSG dosage increased from 5–60 g/L. This may be in regards to the increment in the number of sorption sites (functional groups) at the adsorbent surface, which increase by increasing the dose of the adsorbent material toward the fixed copper ion concentration. These functionalized chemical groups of MANSG were important in the formation of van der Waals bonding with copper ions. Therefore, the additional functional groups inside the magnetic silica gel hybrid played an essential role in metal ions’ binding during the adsorption process, in addition to the adsorption properties of immobilized nano-magnetite onto the silica gel. The optimum dosage of MANSG may be considered as 20 g/L [22,23].

**Figure 11:**
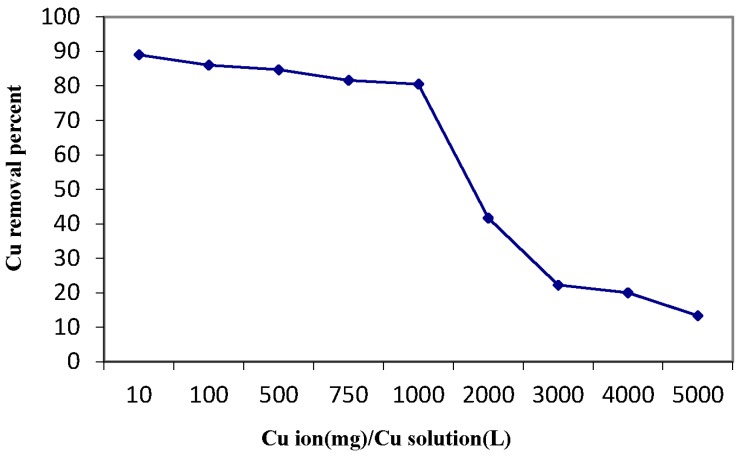
Effect of initial copper ion concentration on the copper sorption process using MANSG (time = 90 min; amount of MANSG = 0.25 g; agitation speed = 200 rpm; solution volume = 25 mL; pH = 7).

The effect of initial metal ion concentration depends on the immediate relation between the concentration of the ion and the available binding sites on an adsorbent surface. It is clear from Figure 11 that the increase in ionic strength of copper solution from 10–1000 ppm using a fixed MANSG amount decreases the magnetic hybrid efficiency from 89% down to 80%. Moreover, a dramatic decline in the percentage of copper decontamination from 80% down to 17% was noticed as the ionic strength of the copper solution increased above 1000 ppm, up to 5000 ppm, using a fixed MANSG amount. This may be due to the saturation of adsorption sites onto the MANSG surface with copper ions; where at a low copper concentration, there will be unoccupied active sites on the magnetic hybrid surface. As the initial copper concentration increases, the active sites required for copper ions’ adsorption will be lacking [22].

**Figure 12:**
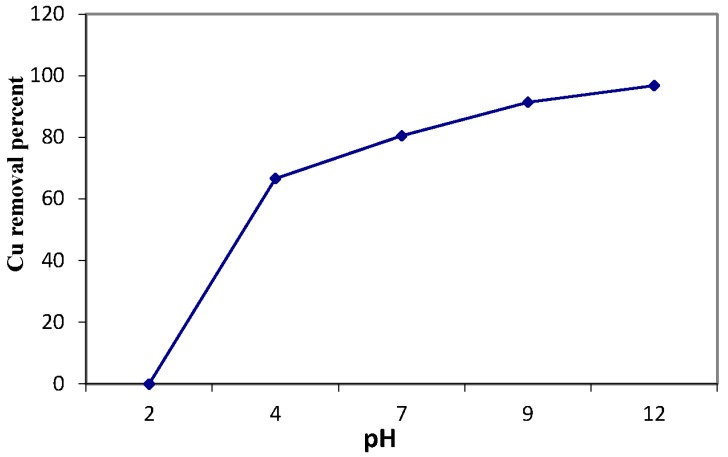
Effect of copper solution pH on the copper sorption process using MANSG (solution concentration of ion = 1000 ppm; amount of MANSG = 0.25 g; agitation speed = 200 rpm; solution volume = 25 mL; time = 90 min).

The acidity of waste solution has two effects on metal ion adsorption onto a specific adsorbent material. Firstly, concerning the acidic media, the protons’ presence in the acidic solutions can protonate the binding sites of the chelating molecules. However, the hydroxide presence in the basic solutions may be complex and precipitates many metal ions [9]. In order to elucidate the influence of this important parameter in the sorption process, the pH of the copper solution was varied over the 2–12 range through the batch procedure. Figure 12 declares that the adsorption percentage of Cu(II) sorption onto MANSG increases with the increase in the pH values for the studied pH range. This may be related to the fact that at a low pH, the MANSG surface tends to be more positive, and electrostatic repulsion with copper cations takes place, inhibiting the sorption process, resulting in decreasing copper sorption onto MANSG. Therefore, the optimum pH values for the maximum Cu(II) were at pH ≥ 7. In order to avoid hydrolyzing, which may cause copper ion precipitation at higher pH values (above seven), pH 7.0 was chosen as the optimum pH for screening the influence of the remaining processing parameters on the sorption process. Moreover, this pH simulates the natural pH of drinking water [24]. This result of the optimum pH value for copper removal onto MANSG was confirmed by many other researchers [9,25].

The effect of agitation speed on the percent of copper removal using MANSG was monitored. The percentage of copper removal seemed to be affected by the agitation speed for values between 0 and 200 rpm, thus confirming that the influence of external diffusion on the copper sorption kinetics plays a significant role; where the increase in the agitation speed decreases the boundary layer resistances, which improve the copper mass transfer into the bulk solution, increasing the copper sorption process. However, as the mixing rate was improved above 200 rpm, the percentage of copper removal was still almost constant. Accordingly, a 200-rpm shaking rate is sufficient to assure that the entirety of the surface binding sites onto MANSG is readily available for copper ions’ uptake and is selected as the optimum mixing speed [24].

**Figure 13:**
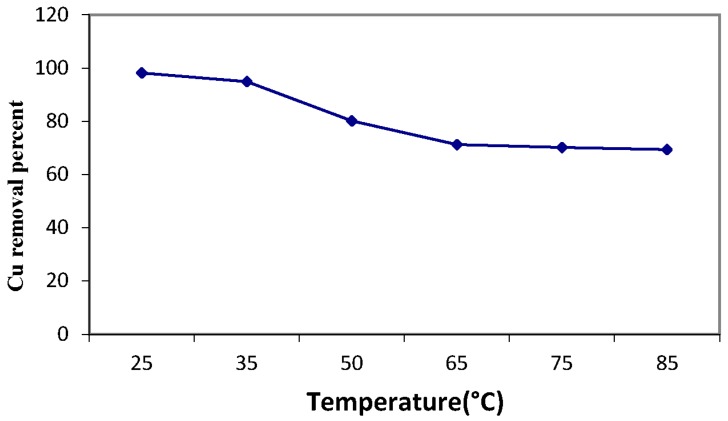
Effect of solution temperature on the copper sorption process using MANSG (solution concentration of ion = 1000 ppm; amount of MANSG = 0.25 g; agitation speed= 200 rpm; solution volume = 25 mL; pH = 7; time = 90 min).

The effect of solution temperature on the copper sorption process is illustrated in Figure 13. This figure displays that the adsorption process of copper ions onto MANSG may be an exothermic process. This sorption behavior may tend to decrease the copper sorption onto MANSG as the solution temperature improved above 25 °C. This decline in the copper sorption with the increment in temperature may decrease the adsorptive forces between the metal ions and the active sites on the MANSG adsorbent surface as a result of decreasing its adsorption capacity [22].

### 3.5. Regeneration of the Magnetic Amine-Functionalized Nano-Silica Gel

**Figure 14:**
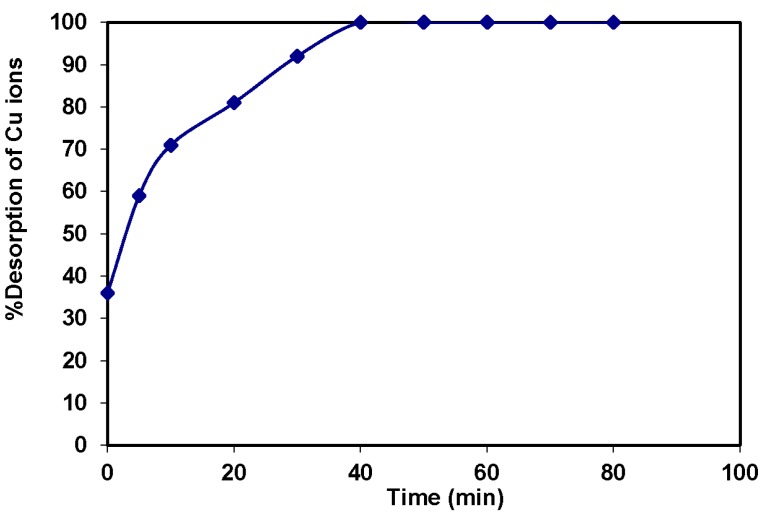
Desorption kinetics of copper ions from MANSG using 0.02 N EDTA.

Above all, magnetic-based nano-adsorbents can be produced at relatively low cost. The high adsorption capacity, low cost, easy separation and regeneration make magnetic-based nano-adsorbents technologically and economically advantageous. The main purpose of the recovery and reuse of magnetic-based nanoparticles is to reduce the overall water treatment process costs and energy consumption. The magnetic properties of these materials facilitate their separation from the treatment media using an external magnetic field after finishing the water treatment process without any requirement for power sources, such as the centrifugational force for material separation. Therefore, the contaminated magnetic amine-functionalized nano-silica gel (MANSG) with the adsorbed copper ions collected after the treatment process was regenerated using EDTA to be reused again as an efficient adsorbing material for the copper sorption process from polluted wastewater. It is indicated by Figure 14 that the percentage of copper desorption was increased with increasing the copper desorption time and reaches almost complete desorption (100%) within 50 min. Accordingly, MANSG was completely activated to be reused as a magnetic adsorbent material within 50 min using 0.02 N EDTA solution; EDTA being a hexadentate chelating ligand that forms a stronger complex with the Cu^2+^ ion compared to the amine group present in the magnetic amine-functionalized silica gel material.

### 3.6. Equilibrium Isotherm Modelling for Copper Decontamination Using Magnetic Amine-Functionalized Nano-Silica Gel

The copper sorption data of MANSG recorded at equilibrium conditions have been analyzed using the linear forms of different equilibrium isotherm models. The applicability of these isotherm equations to the sorption systems was compared by judging the correlation coefficients, *R*^2^ [26].

#### 3.6.1. Langmuir Isotherm

The linear form of the Langmuir isotherm is presented in Equation (3):

(3) $$\frac{C_{e}}{q_{e}}=\frac{1}{q_{m}}C_{e}+\frac{1}{q_{m}b}$$

where q_e_ is the amount of solute adsorbed per unit weight of adsorbent at equilibrium (mg/g), *C_e_* the equilibrium concentration of the solute in the bulk solution (mg/L), q_m_ the maximum adsorption capacity (mg/g) and b the constant related to the free energy of adsorption (L/mg) [27].

**Figure 15:**
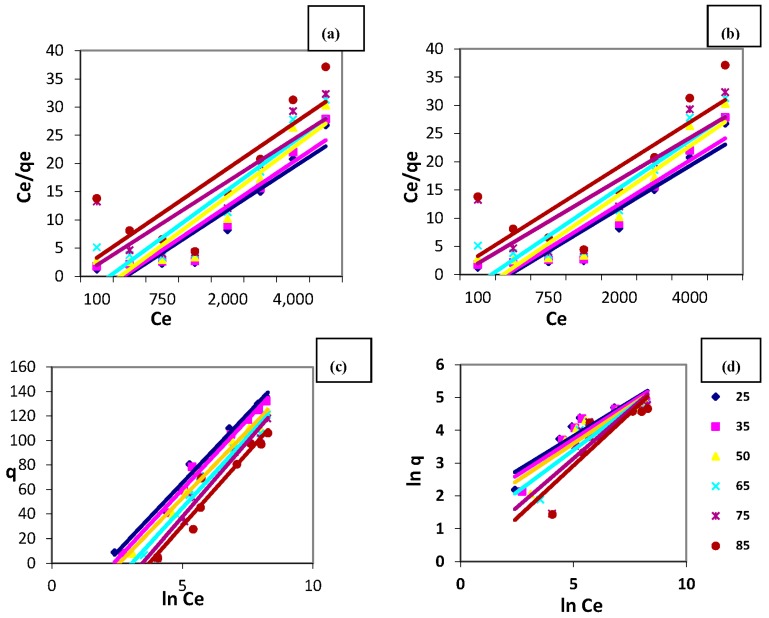
Equilibrium isotherm models for the copper sorption process at different solution temperatures (amount of MANSG = 0.25 g; agitation speed = 200 rpm; solution volume = 25 mL; pH = 7): (**a**) Langmuir isotherm model; (**b**) Freundlich isotherm model; (**c**) Temkin isotherm model; (**d**) Elovich isotherm model.

The adsorption data for copper ions onto MANSG at equilibrium were analyzed to fit the linearized expression of the Langmuir isotherm model that is investigated in Figure 15a. The calculated correlation coefficient value of this model was ranged between *R*^2^ = 0.9981-0.9223 indicates that there is strong positive evidence that the adsorption of Cu onto MANSG follows the Langmuir isotherm. Accordingly, the copper sorption process is suggested to take place as mono-layer adsorption onto the fabricated magnetic hybrid. These results confirm the amine functionality of silica gel; where, the mono-layer adsorption of copper gives the prediction of some sort of chemical reaction happening between the adsorbent matrix and copper ions [28]. Therefore, it can be concluded that the copper ions’ decontamination using MANSG takes place through the chelation process. Langmuir parameters for copper ions sorption, *q_m_* and *b*, were calculated from the slope and intercept of this figure and are tabulated in Table 1. It was indicated from this table that the maximum mono-layer adsorption capacity of 140.845070 mg/g was recorded at room temperature (25 °C). Moreover, Table 1 evidences that the increase in the solution temperature is associated with decline of the value of the maximum mono-layer adsorption capacity (*q_m_*). These results confirm the exothermic nature of the copper sorption process onto MANSG.

The essential characteristics of the Langmuir isotherm are defined by a dimensionless separation factor, *R_L_*, which is indicative of the isotherm shape that predicts whether an adsorption system is favorable or unfavorable. *R_L_* is defined as [27]:

(4) $$R_{L}=\frac{1}{1+bC_{O}}$$

where (*C_o_*) is the initial concentration of copper ions in the solution; the calculated values of *R_L_* are tabulated in Table 1. The calculating values of *R_L_* for copper ions’ sorption using MANSG showed favorable adsorption, since the *R_L_* values fall between zero and one [29]. This again confirms that the Langmuir isotherm was favorable for the sorption of copper ions onto MANSG under the conditions used in this study.

#### 3.6.2. Freundlich Isotherm

The Freundlich isotherm is an empirical equation that encompasses the heterogeneity of sites and the exponential distribution of sites and their energies. The sorption data have been analyzed using the logarithmic form of the Freundlich isotherm as shown below:

(5) $$\ln\;q_{e}=\ln\;K_{f}+\frac{1}{n}\ln\;C_{e}$$

where *K_F_* and *n* are Freundlich constants related to adsorption capacity and adsorption intensity, respectively; the constant *K_F_* is related to the sorption capacity of the sorbent material, and the parameter 1/*n* is indicative of the intensity of the adsorption between the adsorbent-adsorbate, calculated in Figure 15b. The inverse of *n* indicates how strongly the copper ions attached to the MANSG surface [26]. The magnitude of the exponent *n* gives an indication of the favorability of adsorption. It is generally stated that the calculated values of *n* ranged from 2–10, which represent good adsorption. However, if the *n* value falls to 1–2, this gives a prediction of the difficulty of the adsorption process. If the *n* value is less than one, this represents poor adsorption characteristics. Regarding our case study that deals with copper sorption onto MANSG, the calculated *n* value was equal to 2.3 at 25 °C (Table 2). Therefore, MANSG represents a good adsorbent for copper ions. However, it is indicated by Table 2 that the calculated n value was decreased with increasing solution temperature, indicating the negative effect of heat on the copper ions’ adsorption process on MANSG [30]. 

Comparing the correlation coefficient values (0.8709–0.7082) of the Freundlich linearity with that of the Langmuir linearity, it can be observed that the Langmuir isotherm model was more suitable to describe the copper sorption process onto MANSG because of its higher correlation coefficient values compared to the Freundlich isotherm [30]. These results give an indication of the prevalence of the mono-layer adsorption of copper ions onto MANSG, rather than the multi-layer adsorption. These results show the vital role of the amine functional group in silica gel in the copper ions’ chelation process rather than physical adsorption onto the magnetic surface.

#### 3.6.3. Temkin Isotherm

The linear form of the Temkin isotherm is shown in Equation (6):

(6) $$q_{e}=B\;\ln\;K_{T}+B\;\ln\;C_{e}$$

where

(7) $$B=\frac{RT}{b}$$

The Temkin isotherm assumes that the adsorption is characterized by a uniform distribution of the binding energies, up to some maximum energy *B*, which is related to the heat of adsorption; *R* is the universal gas constant (J/mol·K); *T* is the temperature (K); *b* is the variation of the adsorption energy (J/mol); and *K_T_* is the equilibrium binding constant (L/mg) corresponding to the maximum binding energy. A plot of *q_e_ vs.* ln*C_e_* (Figure 15c) enables the determination of the isotherm constants *B* and *K_T_* from the slope and the intercept, respectively [28]. The adsorption data for copper ions onto MANSG at equilibrium were analyzed by a regression analysis to fit the Temkin isotherm model; the higher values of the correlation coefficient that ranged between 0.9868 and 0.9258 show a good linearity. Therefore, the Temkin isotherm model can describe the copper adsorption process onto MANSG [30]. The calculated Temkin isotherm parameters are listed in Table 3. The variation in the adsorption energy (*b*) is positive over the studied temperature range, which confirms that the copper adsorption reaction onto MANSG is exothermic [30].

#### 3.6.4. Elovich Isotherm

The linear form of the Elovich isotherm is shown in Equation (8):

(8) $$\ln\;\frac{q_{e}}{C_{e}}=\ln(K_{E}\times q_{m})-\frac{q_{e}}{q_{m}}$$

**Figure 16:**
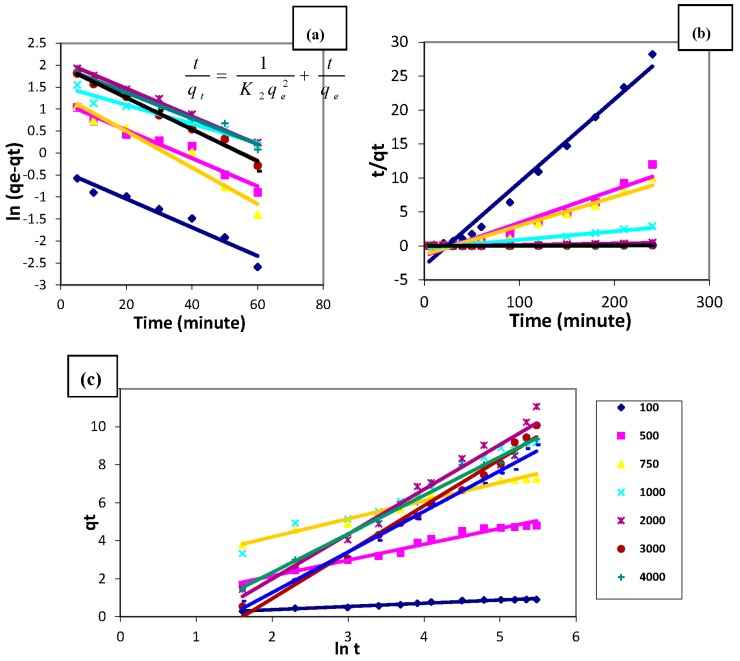
Kinetic models for the copper sorption process (solution temperature = 25 °C; amount of MANSG = 0.25 g; agitation speed = 200 rpm; solution volume = 25 mL; pH = 7): (**a**) pseudo-first-order rate model; (**b**) pseudo-second-order rate model; (**c**) the simple Elovich model.

The Elovich isotherm constants, *K_E_* and *q_m_*, as well as the correlation coefficients for the copper ions’ adsorption system using MANSG are obtained from Figure 16d. The value of the maximum adsorption capacity (*q_m_*) determined using the linear transformation of the Elovich equation is equivalent to 39.6825 mg/g, which is much lower than that estimated from the Langmuir equation (140.845 mg/g) at ambient temperature, as shown in Table 4. This means that the assumption of the exponential covering of adsorption sites that implies multilayer adsorption is not accepted. This result gives the predication that the copper ion sorption process takes place through mono-layer sorption onto MANSG, confirming that the process is mainly chelation and confirming the previous results. Therefore, the Elovich model is not suitable to describe the adsorption of copper ions onto MANSG [30]. Furthermore, Table 4 illustrates that the values of the correlation coefficients are not high enough to confirm the linearity of the Elovich equation.

#### 3.6.5. Dubinin-Radushkevich Isotherm

The Langmuir and Freundlich isotherms are insufficient to explain the physical and chemical characteristics of the adsorption process. The Dubinin-Radushkevich (D-R) isotherm is commonly used to describe the sorption isotherms of single solute systems. The D-R isotherm, apart from being the analogue of the Langmuir isotherm, is more general than the Langmuir isotherm, as it rejects the homogeneous surface or constant adsorption potential. The D-R isotherm is expressed as [31]:

(9) $$\ln\;q_{e}=\ln\;q_{m}-\beta \varepsilon^{2}$$

where

(10) $$\varepsilon =RT\ln(1+\frac{1}{C_{e}})\;\text{a}\;=\;1$$

where *q_e_* is the amount of metal ions adsorbed per unit weight of MANSG (mg/g), *q_m_* is the maximum sorption capacity (mg/g), *β* is the activity coefficient related to adsorption mean energy (mol^2^/kJ^2^) and *ε* is the Polanyi potential described as a function of *C_e_* that represents the equilibrium concentration of the Cu(II) in solution (mg/L), *R* is the gas constant (8.314 J/mol K) and *T* is the temperature [32].

The D-R isotherm model is applied to the equilibrium data obtained from the empirical studies for copper ions’ removal using MANSG to determine the nature of the sorption processes whether it is physical or chemical. 

A plot of ln*q_e_* against *ε*^2^ (figure not investigated) yields a straight line with *R*^2^ values ranging between 0.8381 and 0.8159 (table not investigated), indicating that the D-R model is less fit to the experimental data compared to the Langmuir and Temkin isotherm models, indicating that the D-R model was not able to describe adequately the relationship between the amount of Cu ions sorbed by MANSG and its equilibrium concentration in the solution [33].

### 3.7. Copper Sorption Kinetic Modelling

#### 3.7.1. Pseudo-First-Order Rate Model

The first-order rate equation of Lagergren is one of the most widely-used ones for the sorption of a solute from liquid solution and is represented as:

(11) $$\ln(q_{e}-q_{t})=\ln\;q_{e}-K_{1}t$$

where *q_e_* and *q_t_* are the amounts of ions sorbed (mg/g) at equilibrium and at time t (min), respectively, and *K*_1_ (min^−1^) is the first-order reaction rate constant. The first-order-rate constant *K*_1_ can be obtained from the slope of the plot ln(*q_e_* − *q_t_*) *vs.* time, as illustrated in Figure 16a. The pseudo-first-order considers the rate of occupation of adsorption sites to be proportional to the number of unoccupied sites. The estimated reaction rate constant and the correlation coefficient (*R*^2^) values of the first-order rate model fitting are reported in Table 5. It was indicated from this table that the correlation coefficients are not high enough for all studied copper concentrations (*R*^2^ ~ 0.93), Furthermore, the estimated values of *q_e_* calculated from the equation differ from the experimental values, which indicate that the kinetics of copper sorption onto MANSG cannot be described by the first-order equation [34].

#### 3.7.2. Pseudo-Second-Order Rate Model

This model is based on the sorption equilibrium capacity and may be expressed in the form:

(12) $$\frac{t}{q_{t}}=\frac{1}{K_{2}q_{e}^{2}}+\frac{t}{q_{e}}$$

where *K*_2_ is the second-order reaction rate equilibrium constant (g/mg min). A plot of *t*/*q_t_* against *t* should give a linear relationship for the applicability of the second-order kinetic. Figure 15b shows the linearized form of the pseudo-second-order model for describing the sorption process of different initial copper ion concentrations onto MANSG. The correlation coefficients (*R*^2^) for the pseudo-second-order rate model and its parameters are investigated in Table 6. The results obtained from applying the second order kinetic model indicated that the correlation coefficient (*R*^2^) values of model fitting are high enough (<0.9745) for all studied copper concentrations to be suitable to describe the kinetics of the copper sorption process. Furthermore, Table 6 declares that the estimated values of *q_e_* calculated from the model equation are not greatly different from the experimental values. These results indicate that the second-order kinetic model is appropriate to describe the copper sorption process onto MANSG [24].

#### 3.7.3. Simple Elovich Model

A widely-used equation to describe the kinetics of the chemisorption of fluid on solids was proposed by Elovich. This model may be expressed in the form:

*q_t_* = *α* + *β* ln *t* (13)

where *α* represents the rate of chemisorption at zero coverage (mg/g min) and *β* is related to the extent of surface coverage and activation energy for chemisorption (g/mg); the plot of *q_t_ vs.* ln *t* should give a linear relationship for the applicability of the simple Elovich kinetic model. Figure 15c illustrates the plot of *q_t_* against ln *t* for the copper ions; sorption at different initial concentrations onto MANSG. It was noticeable from the correlation coefficient values of Elovich linearization that this model is applicable for describing the copper sorption process onto MANSG; this suggests that the sorption system studied is chemisorption [28]. This confirms that the copper removal process onto MANSG is taking place mainly through the chelation process.

## 4. Conclusions

The influence of the chemical modification for the activated silica gel on the copper ion sorption capacity was monitored and compared to identify the optimum modification conditions. The copper sorption rate is positively influenced by the improvement in reaction time, reaction temperature and the ratio of silica gel to the chemical reactant concentrations. The most proper amine-functionalized silica gel was immobilized with nano-magnetite using the co-precipitation technique to attain magnetic amine-functionalized nano-silica gel (MANSG). The physical and chemical properties of the fabricated magnetic composite matrix were compared to the properties of the parent silica gel. It was evident from SEM images that both the chemical functionalization and magnetite immobilization processes reduce the silica gel particle diameter from the micro-scale (20 µm) to the nano-scale (50 nm). The copper sorption affinity was enhanced to 98% using MANSG compared to 10% for raw silica gel without modification. The rate of copper sorption onto the synthesized MANSG was negatively affected by both the initial copper concentration and solution temperature. Accordingly, the copper sorption process onto MANSG was identified as an exothermic and follows pseudo-second-order kinetics. On the other hand, the equilibrium of the copper sorption process onto MANSG obeys the Langmuir model, which confirms the process was controlled mainly by the chelation process as a mono-layer. According to the recorded results, the present study confirms that MANSG, which is characterized by its magnetic properties, can be used for the decontamination of waste solution polluted by copper ions with a decontamination percentage equal to 98%.

## Figures and Tables

**Table 1:** Estimated parameters of the Langmuir isotherm for copper sorption onto MANSG at different solution temperatures.

Solution Temperature (°C)	*q_m_* (mg/g)	*b* (L/mg)	*R*^2^	*R_L_*
25	140.85	0.0052	0.9981	0.951–0.037
35	136.99	0.0042	0.9981	0.957–0.043
50	126.58	0.0039	0.9943	0.962–0.049
65	122.21	0.0026	0.9875	0.975–0.072
75	115.135	0.0014	0.9028	0.986–0.123
85	109.87	0.001	0.9223	0.988–0.142

**Table 2:** Estimated parameters of the Freundlich isotherm for copper ions’ sorption onto MANSG at different solution temperatures.

Solution Temperature (°C)	*K_f_*	*n*	*R*^2^
25	5.55	2.37	0.88
35	4.6	2.27	0.86
50	3.74	2.2	0.82
65	2.31	1.99	0.8
75	1.18	1.68	0.71
85	0.76	1.57	0.76

**Table 3:** Estimated parameters of Temkin isotherm for copper ions sorption onto MANSG at different solution temperatures.

Solution Temperature (°C)	*K_T_* (L/mg)	*B*	*b* (J/mol)	*R*^2^
25	0.12	22.497	0.11	0.99
35	0.09	22.794	0.11	0.99
50	0.08	21.91	0.12	0.98
65	0.05	23.192	0.12	0.97
75	0.03	24.601	0.12	0.95
85	0.02	23.875	0.12	0.93

**Table 4:** Estimated parameters of the Elovich isotherm for the adsorption of copper ions onto MANSG at different solution temperatures.

Solution Temperature (°C)	*K_e_* (L/mg)	*q_m_* (mg/g)	*R*^2^
25	0.04	39.68	0.89
35	0.023	41.67	0.88
50	0.02	42.37	0.8
65	0.01	51.55	0.69
75	0.003	76.92	0.35
85	0.002	85.47	0.33

**Table 5:** Estimated kinetic parameters for the first-order rate kinetic model at different copper concentrations.

Initial Concentration of Cu Ion (ppm)	*R*^2^	*K*_1_ (min^−1^)	*q_e_* (cal.) (mg/g)	*q_e_* (exp.) (mg/g)
100	0.93	0.025	0.616	0.86
500	0.95	0.032	3.194	4.52
750	0.95	0.041	3.758	6.667
1000	0.94	0.021	4.559	8.05
2000	0.99	0.03	7.798	8.333
3000	0.98	0.037	7.53	6.667
4000	0.97	0.03	7.464	8
5000	0.95	0.037	7.716	6.663

**Table 6:** Estimated kinetic parameters of the second-order rate kinetic model at different copper concentrations.

Initial Concentration of Cu Ion (ppm)	*R*^2^	*K*_2_ (g/mg·min)	*q_e_* (cal.) (mg/g)	*q_e_* (exp.) (mg/g)
100	0.98	0.0051	0.82	0.86
500	0.98	0.0016	2.06	4.52
750	0.98	0.0014	2.36	6.67
1000	0.98	0.0092	8.00	8.05
2000	0.97	0.0005	8.85	8.33
3000	0.99	0.0062	6.67	6.67
4000	0.99	0.0575	7.4	8
5000	0.99	0.0001	6.33	6.66
